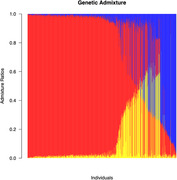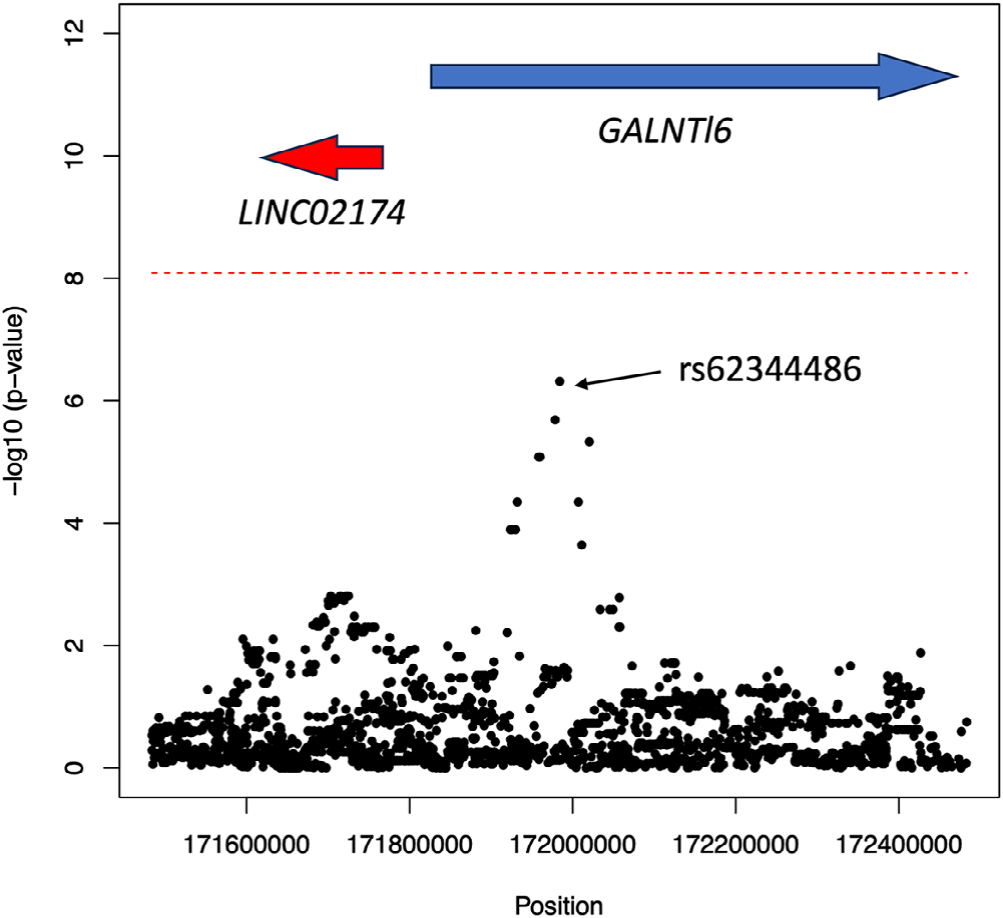# A genome‐wide association study for clinical dementia phenotypes in an ethnoracially diverse cohort

**DOI:** 10.1002/alz.089929

**Published:** 2025-01-09

**Authors:** Shraddha Sapkota, Marcio A Almeida, Lee‐Way Jin, Sarah Tomaszewski Farias, Charles Decarli

**Affiliations:** ^1^ University of California, Davis, Davis, CA USA; ^2^ University of Texas Rio Grande Valley, Brownsville, TX USA; ^3^ University of California Davis Medical Center, Davis, CA USA; ^4^ University California, Davis, Davis, CA USA; ^5^ University of California, Davis, CA USA

## Abstract

**Background:**

Genome‐wide association studies (GWAS) for established clinical dementia phenotypes is often limited to Caucasians from European ancestry. Although the incidence of dementia is higher in African Americans and Hispanics, the inclusion of these groups in GWAS research is less common. We examine GWAS for clinical dementia phenotypes in an ethoracially diverse cohort of older adults.

**Method:**

We used an older‐adult cohort from the University of California, Davis‐Alzheimer’s Disease Research Center (n=1014; mean age= 74.54 (7.50) years; 59.6% female). Participants were diagnosed as cognitively normal (CN; n=329), Mild Cognitive Impairment (MCI; n=448) and Demented (n=234) at final visit. Samples were genotyped and imputed using 1000 genomes as reference. Genetic admixtures components were calculated assuming three original populations resulting in a set of 6078198 high‐quality imputed single nucleotide polymorphisms (SNP) from 1000 participants. Our outcome tested GWAS across clinical dementia phenotypes (CN, MCI, Demented). We further stratified our genetic dataset into non‐synonymous and non‐synonymous high‐deleterious genetic variants. Finally, we performed a replication analysis of the top Alzheimer’s disease‐related genetic risk loci identified in the literature. All genetic association analyses included age, sex, admixture components and education as covariates.

**Result:**

Admixture analysis detected primarily European/Caucasian genetic ancestry and African and Amerindian minorities (Figure 1). We did not observe significant associations for any panel (overall, non‐synonymous, high‐deleterious). Some findings were of interest. Our top genetic risk loci associated with clinical dementia phenotype in the (1) overall panel was rs62344486 (p<4.84e^‐07^) (Figure 2) known to play a protective role against cognitive decline, (2) non‐synonymous panel was rs17832998 in the DACT1 gene (p<1.0e^‐05^), and (3)non‐synonymous high‐deleterious panel were rs41267154 (p<3.4e^‐03^) and rs72697000 (p<1.5e^‐03^) in the FLG gene. We also identified rs17668923 located downstream of TMEM106B gene (p<2.8e^‐05^) and rs56220430 (p<1e^‐04^) near SIGLEC11 gene from our literature review.

**Conclusion:**

We identified two novel SNPs near TMEM106B and SIGLEC11 genes which have been associated with frontotemporal dementia and immunosuppressive signals, respectively. Preliminary GWAS for clinical dementia phenotypes in diverse populations may (1) identity novel genetic risk loci underlying the dynamic neurobiological underpinnings across the dementia continuum and (2) be further validated in larger minority population studies.